# PheWAS-Based Systems Genetics Methods for Anti-Breast Cancer Drug Discovery

**DOI:** 10.3390/genes10020154

**Published:** 2019-02-18

**Authors:** Min Gao, Yuan Quan, Xiong-Hui Zhou, Hong-Yu Zhang

**Affiliations:** 1 Hubei Key Laboratory of Agricultural Bioinformatics, College of Informatics, Huazhong Agricultural University, Wuhan 430070, China; gm@webmail.hzau.edu.cn (M.G.); zhouxionghui@mail.hzau.edu.cn (X.-H.Z.); zhy630@mail.hzau.edu.cn (H.-Y.Z.); 2 School of Computer Science and Technology, Harbin Institute of Technology Shenzhen Graduate School, Shenzhen 518055, China; 3 Lab of Epigenetics and Advanced Health Technology, Space Institute of Southern China, Shenzhen 518117, China

**Keywords:** PheWAS, drug discovery, breast cancer, systems genetics

## Abstract

Breast cancer is a high-risk disease worldwide. For such complex diseases that are induced by multiple pathogenic genes, determining how to establish an effective drug discovery strategy is a challenge. In recent years, a large amount of genetic data has accumulated, particularly in the genome-wide identification of disorder genes. However, understanding how to use these data efficiently for pathogenesis elucidation and drug discovery is still a problem because the gene–disease links that are identified by high-throughput techniques such as phenome-wide association studies (PheWASs) are usually too weak to have biological significance. Systems genetics is a thriving area of study that aims to understand genetic interactions on a genome-wide scale. In this study, we aimed to establish two effective strategies for identifying breast cancer genes based on the systems genetics algorithm. As a result, we found that the GeneRank-based strategy, which combines the prognostic phenotype-based gene-dependent network with the phenotypic-related PheWAS data, can promote the identification of breast cancer genes and the discovery of anti-breast cancer drugs.

## 1. Introduction

Breast cancer is the most common form of cancer and the second leading cause of cancer-related deaths among women worldwide. Breast cancer has no real low-risk population and is a typical global cancer. Approximately 1 million to 1.3 million breast cancer cases are diagnosed every year worldwide [1]. Breast asymmetry, height, family history of breast cancer, age at menarche, parenchyma type, and menopausal status are significant influencing factors of breast cancer [2]. It is very meaningful to explore effective anti-breast cancer drugs.

The past decade has witnessed unprecedented progress in biotechnology, particularly in omics technologies. However, these great technical advances have made limited contributions to the advancement of the pharmaceutical industry. The pharmaceutical drug development process still requires $2.6 billion (estimated by the Tufts Center) and approximately 13.5 years, on average, for a new molecular entity (NME) drug to enter the market [3]. Therefore, we urgently need methods that can efficiently synthesize and utilize biological big data to streamline the drug discovery pipeline.

Because genetic disease genes are important sources for drug targets, medical genetics has found important applications in drug discovery [4,5,6,7]. Currently, a large amount of genetic data has accumulated, particularly in the genome-wide identification of disorder genes. Genome-wide association studies (GWASs) have identified genetic variants that modulate human diseases; many of these associations require further study to replicate the results [8]. On the other hand, the emergence of genetic data coupled to longitudinal electronic medical records (EMRs) offers the possibility of phenome-wide association studies (PheWASs) of disease–gene associations [9]. Denny et al. demonstrated that for many of the GWAS-derived single nucleotide polymorphisms (SNPs), PheWASs were able to rediscover the expected SNP–disease associations while also identifying novel associations [6,9]. Majid et al. proposed that PheWAS results may also provide new opportunities to identify candidates for drug repositioning. Their research shows that 4.9% of drug–disease associations that were derived from traditional PheWASs are supported by clinical evidence [6]. However, because the gene–disease links that were identified by high-throughput techniques are usually too weak to have biological significance, how to use these data efficiently for pathogenesis elucidation and drug discovery is still a challenge.

Systems genetics is a thriving area of study that aims to understand genetics interactions on a genome-wide scale [10]. The HotNet diffusion-oriented subnetworks (HotNet2) algorithm and the GeneRank algorithm are two representative systematic genetic methods. Therefore, it is of great interest to investigate whether the HotNet2 algorithm and the GeneRank algorithm can promote the identification of breast cancer-associated genes from original PheWAS data. In this study, we established two strategies for identifying disease–gene relationships based on the two methods above. We used these two strategies in the screening of anti-breast cancer drugs, with the aim of alleviating the limitations of high-throughput sequencing data.

## 2. Materials and Methods

### 2.1. Data Sets

In this study, PheWAS data were derived from work by Denny et al. [8], which included 522 breast cancer-associated single nucleotide polymorphisms (SNPs). According to the SNP-to-gene mapping procedure used by Nelson et al. [11], 1742 potential breast cancer genes were identified from PheWAS data (Table S1).

The breast cancer-related genes were obtained from eight databases, including the Genetic Association Database (GAD, https://geneticassociationdb.nih.gov/) [12], the Online Mendelian Inheritance in Man (OMIM, http://omim.org/) [13], Clinvar (http://www.ncbi.nlm.nih.gov/clinvar/) [14], Orphanet (http://www.orpha.net/consor/cgi-bin/index.php), GWASdb (http://jjwanglab.org/gwasdb) [15], the NHGRI GWAS Catalog (https://www.ebi.ac.uk/gwas/) [16], and RegulomeDB (http://www.regulomedb.org/) [17], and partial data from the Human Gene Mutation Database (HGMD, http://www.hgmd.cf.ac.uk/ac/index.php) appeared in the work by Wang et al. [18]. A natural language processing tool, MetaMap, was used to standardize disease descriptions of disease genes in these eight databases [19].

Information regarding the association between chemical agents and their corresponding targets was obtained from the Drug–Gene Interaction database (DGIdb, http://dgidb.genome.wustl.edu/) [20], the Therapeutic Target Database (TTD, http://bidd.nus.edu.sg/group/cjttd/) [21], and DrugBank (http://www.drugbank.ca/) [22]. Only clinical activity annotations of the agents recorded in TTD, DrugBank, and ClinicalTrials (http://clinicaltrials.gov/) were used in this work. The clinical activities of agents were also standardized using MetaMap.

### 2.2. HotNet2 Algorithm

HotNet2 is based on an insulated heat diffusion kernel algorithm that considers the heats (reflecting genetic importance) of individual genes as well as the topology of gene–gene interactions [23]. During HotNet2 calculations, the negative logarithms of *p*-values of PheWAS-derived SNPs were used as initial heat vectors for 1742 corresponding disease genes. According to the original literature of the HotNet2 algorithm, the protein–protein interaction (PPI) network was also obtained from the HINT network, iRefIndex, and Multinet [23]. Previously used parameters and procedures for HotNet2 calculations (https://github.com/raphael-group/hotnet2) were applied in this study [23].

### 2.3. Gene Dependency Network

Zhou et al. proposed a method to construct a gene dependency network in 2014, which integrated phenotype information with gene expression data to identify gene dependency pairs by using the method of conditional mutual information [24]. Therefore, the gene dependency network has the advantage of identifying gene dependency relations in the process of cancer prognosis. In this work, we used this network to discover important pathogenic genes associated with phenotypes.

### 2.4. GeneRank Algorithm

The GeneRank algorithm is a method proposed by Morrison et al. in 2005 to reorder genes by combining gene expression information with a network structure derived from gene annotations (gene ontology) or expression profile correlations [25]. The original GeneRank was applied to indirect networks. Here, we used a strategy similar to Zhou et al. to extend it to the directed network [26]. GeneRank’s modification algorithm can be described as follows:

(1) $$r_{j}^{n}=\left(1-d\right)f_{i}+d\overset{N}{\underset{i=1}{\sum }}\frac{w_{ij}r_{i}{}^{n-1}}{deg_{i}}$$

The information from the biological network (we used a gene dependency network here) can be stored in an adjacency matrix, where $w_{ij}$ = 1 if gene *i* is significantly dependent on gene *j* and $w_{ij}$ = 0 otherwise; $deg_{i}$ describes how many nodes are dependent on gene *i*, that is, the out-degree of gene *i*; N is the number of genes in the network; $r_{j}^{n}$ represents the score of gene *j* after *n* iterations and $r_{i}^{n-1}$ is the score of gene *i* after *n* − 1 iterations; $f_{i}$ is the initial score of gene *j*. Here, we set the initial score of gene *j* as the negative logarithm of the *p*-value of the PheWAS-derived gene; *d* (0 ≤ *d* < 1) weighted the gene dependency network. To determine the initial importance of PheWAS and the weight of the gene-dependent network for each half, we set *d* = 0.50 in this work. The iteration of the algorithm stopped when ε < 0.00001, while ε is one-norm of $\left|r_{j}^{n}-r_{j}^{n-1}\right|$.

### 2.5. Enrichment Analysis

The clusterprofiler package in R [27] was used for KEGG (Kyoto Encyclopedia of Genes and Genomes) and GO (Gene Ontology) functional analysis (biological processes) on the top 100 genes in each gene list. The *p*-value adjusted by FDR (False Discovery Rate) (*p*.adjust) < 0.05 was used as the cutoff criterion.

A Kolmogorov–Smirnov test was applied to test whether the drug targets of known drugs with anti-breast cancer activity were enriched on the top of the gene list after PheWAS-Rank analysis.

## 3. Results and Discussion

### 3.1. Identification of Breast Cancer-Associated Genes by HotNet2

In this study, we primarily used the HotNet2 algorithm to calculate the subnetworks of breast cancer based on 1742 PheWAS-derived breast cancer genes. As a result, significant subnetworks of 227 genes were successfully identified from the original PheWAS data (Table S2).

**Figure 1:**
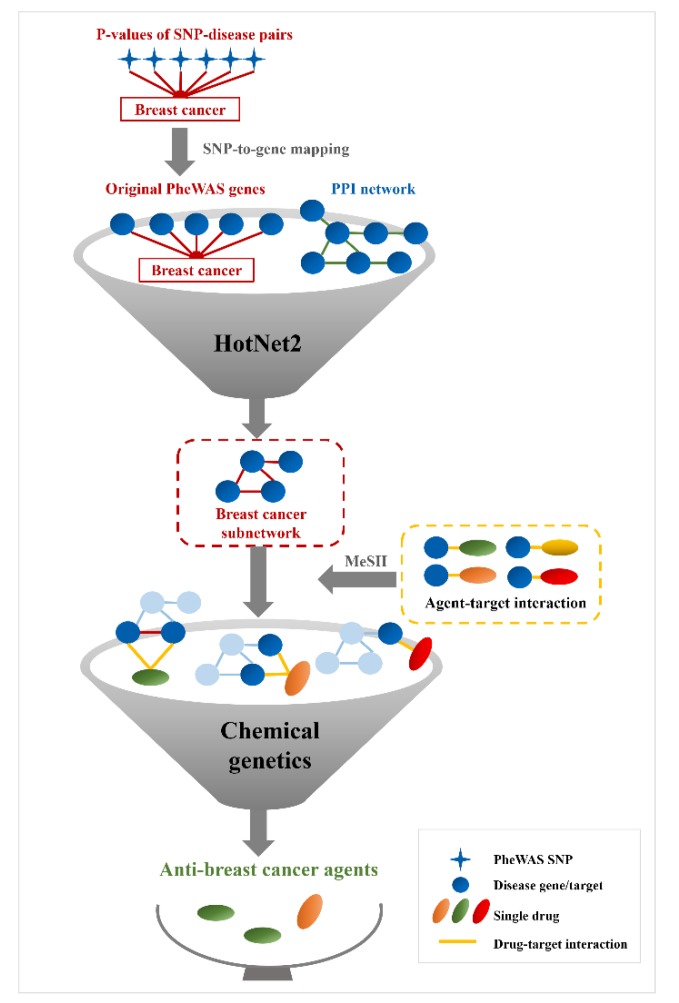
Pipeline of HotNet2-based anti-breast cancer drug discovery. A total of 522 breast cancer-associated single nucleotide polymorphisms (SNPs) were derived from the phenome-wide association study (PheWAS) [9]. The strongly linked variants of these SNPs were obtained by linkage disequilibrium (LD) analysis on the basis of the 1000 Genomes Project (r^2^ ≥ 0.8). Then, the genes potentially regulated by the PheWAS-derived loci were identified through the combinatorial application of various information, such as physical proximity to the gene, gene expression quantitative trait loci (eQTL), and the locations of variants overlapped with DNase I-hypersensitive site (DHS) peaks. Finally, a total of 1742 breast cancer-associated genes were identified from the PheWAS data. After HotNet2 calculation, significant subnetworks including 227 genes were successfully identified from the original PheWAS data. Finally, these agents that target HotNet2-derived pathogenic genes were predicted to be potential anti-breast cancer drugs. PPI: protein–protein interaction. MeSH: Medical Subject Headings.

Next, to validate the effectiveness of the breast cancer-associated genes, which were included in the significant subnetworks that were identified by HotNet2, we obtained 2841 breast cancer-related genes from eight disease gene databases (Materials and Methods). Of 1742 original PheWAS-derived genes, 208 (11.94%) were breast cancer-related genes that were documented in these databases (Table S1). For 227 HotNet2-identified breast cancer genes, this ratio rose to 19.38% (44 of 227) (Table S2), which is significantly higher than the non-HotNet2-identified genes that were obtained from the original PheWAS-derived genes (164 of 1515, 10.83%) (*p* = 2.10 × 10^−4^, Chi-squared test). These results indicate that the breast cancer-related genes that were identified by HotNet2 are effective and worthy of further use for anti-breast cancer drug prediction (Figure 1).

### 3.2. Anti-Breast Cancer Drug Discovery Based on HotNet2-Identified Genes

Using the information on chemical agent–target associations and clinical activity annotations of the agents (Materials and Methods), we evaluated the performance of HotNet2 methods in identifying clinically validated agents. Based on the HotNet2-identified genes, we obtained 242 potential anti-breast cancer agents. A total of 7.44% (18 of 242) of these agents were supported by clinical tests (Table S3)—fewer than the original PheWAS-derived agents (88 of 894, 9.84%). The above results indicated that the HotNet2 algorithm is indeed useful for identifying breast cancer-associated genes. However, the performance of this method may fundamentally depend on the quality of the PPI and the initial heat vectors, and the latter denotes the strength of the gene–disease association. Therefore, the low quality of the original PheWAS-derived gene–disease associations may be one of the reasons for the poor effectiveness of HotNet2 in anti-breast cancer drug discovery. To overcome the limitations of the HotNet2 algorithm, we need to try other systems genetics methods for the discovery of anti-breast cancer drugs.

### 3.3. Identification of Breast Cancer-Associated Genes by PheWAS-Rank

**Figure 2:**
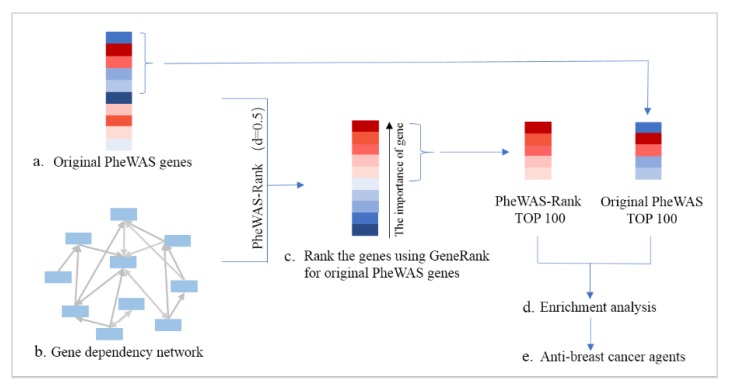
Pipeline of GeneRank-based anti-breast cancer drug discovery. Based on the 1742 PheWAS-identified breast cancer-related genes (**a**), we combined the gene-dependent network (**b**) to rank the original PheWAS data using the GeneRank algorithm (**c**). To cause the topology of the biological network and the original PheWAS to have the same weight, we set *d* = 0.5. Then, we performed a series of enrichment analyses on the original PheWAS (PheWAS-Rank gene set) and PheWAS-Rank top 100 genes (PheWAS-Rank gene set) (**d**). Finally, the agents that target the PheWAS-Rank gene set were predicted to be potential anti-breast cancer drugs (**e**).

The GeneRank algorithm concerns both the topological structure of the biological network and the importance of the nodes in the network, and it is more helpful for identifying the truly important genes that are associated with the disease. We used the negative logarithms of the *p*-values of PheWAS-derived genes as the initial importance of the node and used the gene-dependency network as the network topological structure, which contains the interdependence of genes that are associated with the prognostic phenotypes in breast cancer, to reorder the importance of genes. We defined this method as the PheWAS-Rank method. After ranking the intersection of the original PheWAS genes and the breast cancer gene dependency network, we obtained a sorted list of genes containing 506 genes (Figure 2; Table S4).

To test whether the PheWAS-Rank-based strategy can improve the performance of detecting important genes in breast cancer, we used the same breast cancer-related genes from eight databases in the same way as the HotNet2-based strategy described above. In this work, the top 100 genes were selected as important genes in the original PheWAS-derived gene list (Table S1) and in the PheWAS-Rank gene list (Table S4), and we defined these two gene lists as the PheWAS gene set and the PheWAS-Rank gene set, respectively (Table S5). Of the PheWAS gene set, 13 genes were breast cancer genes recorded in the eight databases, while 36 genes from the PheWAS-Rank gene set were breast cancer genes that were recorded in the eight databases (Table S4), which is significantly higher than the original PheWAS-derived genes (*p* = 1.56 × 10^−4^, Chi-squared test) (Table S5). This result suggests that the GeneRank-based strategy combined with the gene dependency network can significantly identify breast cancer-related genes.

**Figure 3:**
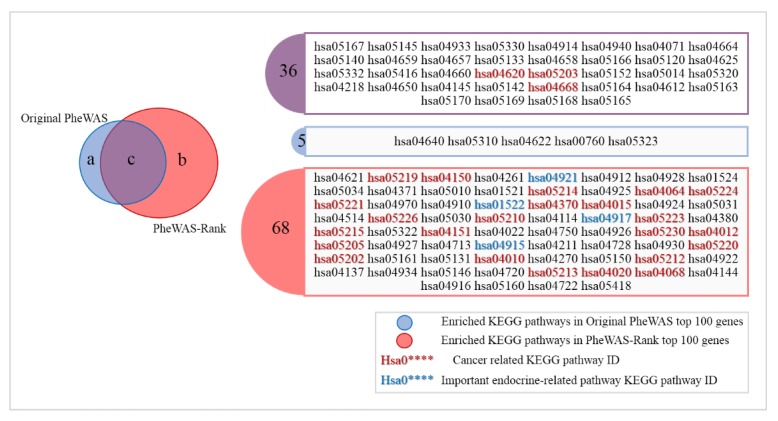
KEGG functional analysis on the top 100 genes of the original PheWAS-derived gene list (Table S1) and the PheWAS-Rank gene list (Table S4) (*p*.adjust < 0.05) (Table S6). The PheWAS gene set enriched 41 KEGG pathways (**a**,**c**); the PheWAS-Rank gene set enriched 104 KEGG pathways (**b**,**c**); there were 36 KEGG pathways that were enriched in both of the gene lists (**c**). KEGG: Kyoto Encyclopedia of Genes and Genomes.

To verify whether the important genes in our PheWAS-Rank gene list are especially related to cancer, we used the clusterprofiler package in R for KEGG and GO functional analyses of the PheWAS gene set and PheWAS-Rank gene set. As a result, 41 and 104 KEGG pathways with an adjusted *p*-value less than 0.05 were significantly enriched within the PheWAS gene set and the PheWAS-Rank gene set, respectively (Table S6). As shown in Figure 3, the PheWAS gene set only enriched 3 cancer-related KEGG pathways, while the PheWAS-Rank gene set enriched 26 cancer-related KEGG pathways, which included many key cancer-related pathways, such as ”breast cancer” (hsa05224), ”TNF signaling pathway” (hsa04668) [28], ”MAPK signaling pathway” (hsa04010) [28], ”VEGF signaling pathway” (hsa04370) [29], ”NF-kappa B signaling pathway” (hsa04064) [28,29], and ”PI3K-Akt signaling pathway” (hsa04151) [28]. In addition, the PheWAS-Rank gene set was also enriched with many endocrine-related pathways that are closely related to breast cancer, such as the ”estrogen receptor pathway” (hsa04915) [30], ”prolactin signaling pathway” (hsa04917) [31], and ”oxytocin signaling pathway” (hsa04921) [32].

**Figure 4:**
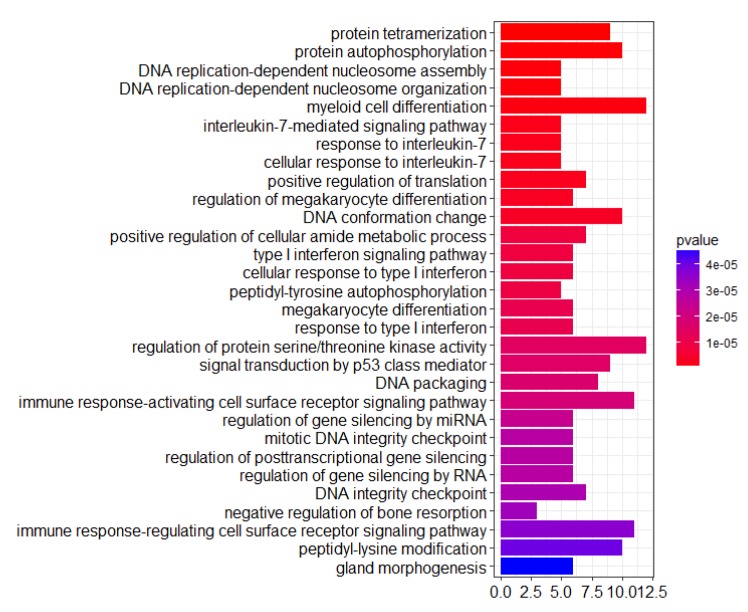
GO functional analysis (biological processes) of the top 100 genes of the PheWAS-Rank gene list (Table S4) (*p*.adjust < 0.05). The abscissa represents the GeneRatio. GO: Gene Ontology.

Unlike the results from the KEGG functional analysis, the PheWAS gene set was not enriched in any annotation in the GO functional analysis, with an adjusted *p*-value less than 0.05, while the PheWAS-Rank gene set was enriched in 193 biological functions (Table S7). From this result, we can see that the PheWAS-Rank gene set was enriched with many functions that are related to cancer, such as cell differentiation, apoptosis, transcriptional regulation, and immune-related functions (Figure 4). The diversity of these identified biological functions suggests that these genes may be involved in different pathways in the process of tumorigenesis. In summary, a conclusion can be drawn that the top genes in the PheWAS-Rank gene list could be enriched for cancer-related functional pathways. We also further explored the application of this strategy in predicting anti-breast cancer drugs.

### 3.4. Anti-Breast Cancer Drug Discovery Based on PheWAS-Rank-Identified Genes

**Figure 5:**
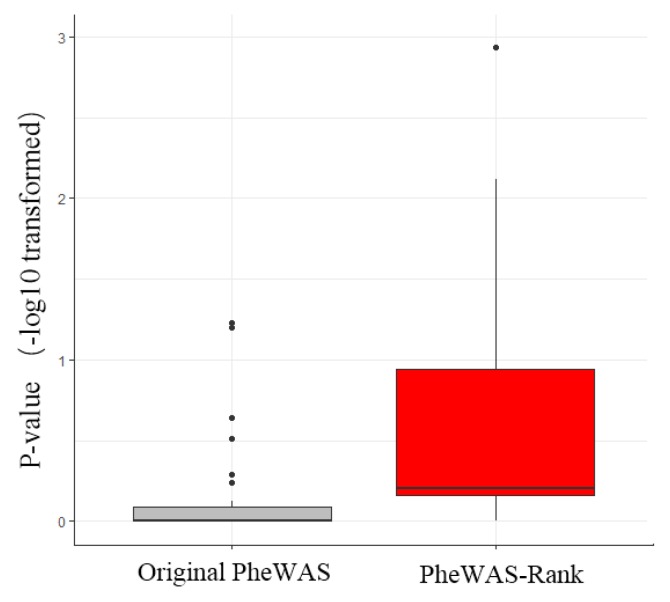
The original PheWAS top 100 gene list and PheWAS-Ranked top 100 gene list was validated with a Kolmogorov–Smirnov test using 63 known anti-breast cancer active drugs.

To verify that the PheWAS-Rank-based strategy contributes to the discovery of anti-breast cancer drugs, we validated the original PheWAS-derived gene list and the PheWAS-Rank gene list with a Kolmogorov–Smirnov test using 63 known anti-breast cancer active drugs (Table S8). The Kolmogorov–Smirnov test showed that the enrichment results of the PheWAS-Rank gene set obtained by our method were significantly better than the enrichment results of the PheWAS gene set (Figure 5). This result indicates that the PheWAS-Rank-based strategy is more helpful for the discovery of anti-breast cancer drugs in the original PheWAS data.

Finally, we used the PheWAS gene set and the PheWAS-Rank gene set to predict anti-breast cancer drugs and tested the results with the drugs that are known to have anti-breast cancer activity recorded in ClinicalTrials. Based on the PheWAS gene set, we obtained 127 potential anti-breast cancer agents, and 3.15% (4/127) of these agents were supported by clinical tests (Table S9). Based on the PheWAS-Rank gene set, we obtained 263 potential anti-breast cancer agents. A total of 12.17% (32/263) of these agents were supported by clinical tests (Table S10), significantly more than the PheWAS-derived agents (*p* = 3.94 × 10^−3^, Chi-squared test).

In our study, the PheWAS-Rank-based strategy was superior to the HotNet2 method in improving the druggability of the agents that target PheWAS-derived genes. These results suggest that the genes that were found based on the PheWAS-Rank strategy are potential genes that are strongly associated with the diseases and are more druggable [7], in that the agents targeting these genes are stronger drug candidates. Hence, this strategy can promote drug repositioning for breast cancer. In fact, in this article, we obtained a collection of 236 potential anti-breast cancer drugs that can be used for further validation of anti-breast cancer activity.

## 4. Conclusions

In summary, in this omics era, we are facing a flood of biomedical data. Integrating high-throughput sequencing technology and genetic approaches has revealed an increasing number of disease-associated variants/genes. Efficiently utilizing these data to find novel drugs to protect human health is a great challenge. In this study, we explored two systems genetics approaches that could establish reliable gene–disease links and then compared their potentials in drug discovery. Since the HotNet2 method relies on the reliability of initial heats for the original genes, the application of this method in drug discovery is limited when the raw data are unreliable. In contrast, the GeneRank-based strategy takes into account the importance of network topology, thus effectively overcoming the shortcomings of the HotNet2 method. In our study, we combined PheWAS data with systems genetics methods for the first time to overcome the weak correlation between PheWAS-derived genes and breast cancer and improve the clinical effectiveness of drug prediction. In addition to breast cancer, with the recent development of high-throughput sequencing, the PheWAS method has accumulated a large number of cancer-related pathogenic genes, and this pipeline can also be used to study other cancers. Moreover, as long as we have the initial genetic importance data of the disease and its corresponding gene-dependency network, we can readily extend this method to other diseases. However, our approach still has some limitations. For instance, this method requires sufficient genotype, phenotype, and prognostic information for the diseases. Therefore, it is not applicable to rare diseases. However, with the rapid accumulation of biomedicine big data, this strategy is expected to find broad applications for navigating in the broad drug space and reach islands that are enriched with promising drugs.

## Supplementary Materials

Supplementary materials can be found at https://www.mdpi.com/2073-4425/10/2/154/s1.
